# Inconsistent Results of Diagnostic Tools Hamper the Differentiation between Bee and Vespid Venom Allergy

**DOI:** 10.1371/journal.pone.0020842

**Published:** 2011-06-15

**Authors:** Gunter J. Sturm, Chunsheng Jin, Bettina Kranzelbinder, Wolfgang Hemmer, Eva M. Sturm, Antonia Griesbacher, Akos Heinemann, Jutta Vollmann, Friedrich Altmann, Karl Crailsheim, Margarete Focke, Werner Aberer

**Affiliations:** 1 Division of Environmental Dermatology and Venerology, Department of Dermatology, Medical University of Graz, Graz, Austria; 2 Department of Chemistry, University of Natural Resources and Applied Life Sciences, Vienna, Austria; 3 Floridsdorf Allergy Center, Vienna, Austria; 4 Institute of Experimental and Clinical Pharmacology, Medical University of Graz, Graz, Austria; 5 Division of Biostatistics, Center for Medical Research, Medical University of Graz, Graz, Austria; 6 Institute of Zoology, University of Graz, Graz, Austria; 7 Institute of Pathophysiology, Medical University of Vienna, Vienna, Austria; Ulm University, Germany

## Abstract

**Background:**

Double sensitization (DS) to bee and vespid venom is frequently observed in the diagnosis of hymenoptera venom allergy, but clinically relevant DS is rare. Therefore it is sophisticated to choose the relevant venom for specific immunotherapy and overtreatment with both venoms may occur. We aimed to compare currently available routine diagnostic tests as well as experimental tests to identify the most accurate diagnostic tool.

**Methods:**

117 patients with a history of a bee or vespid allergy were included in the study. Initially, IgE determination by the ImmunoCAP, by the Immulite, and by the ADVIA Centaur, as well as the intradermal test (IDT) and the basophil activation test (BAT) were performed. In 72 CAP double positive patients, individual IgE patterns were determined by western blot inhibition and component resolved diagnosis (CRD) with rApi m 1, nVes v 1, and nVes v 5.

**Results:**

Among 117 patients, DS was observed in 63.7% by the Immulite, in 61.5% by the CAP, in 47.9% by the IDT, in 20.5% by the ADVIA, and in 17.1% by the BAT. In CAP double positive patients, western blot inhibition revealed CCD-based DS in 50.8%, and the CRD showed 41.7% of patients with true DS. Generally, agreement between the tests was only fair and inconsistent results were common.

**Conclusion:**

BAT, CRD, and ADVIA showed a low rate of DS. However, the rate of DS is higher than expected by personal history, indicating that the matter of clinical relevance is still not solved even by novel tests. Furthermore, the lack of agreement between these tests makes it difficult to distinguish between bee and vespid venom allergy. At present, no routinely employed test can be regarded as gold standard to find the clinically relevant sensitization.

## Introduction

Personal history, skin testing, and detection of sIgE, are the mainstays of the diagnostic procedure in cases of hymenoptera venom allergy. Although sensitization to both, honeybee and vespid venom, is observed in up to 59% of patients [1], clinically relevant double sensitization (DS) is rare and patients usually react either to bee or to wasp stings. Therefore, in clinical routine it can be sophisticated to find the relevant venom for specific immunotherapy with common diagnostic tests.

There are several reasons for DS: Generally, a true DS with antibodies to different bee and vespid venom allergens should be considered. DS can also be a result of an around 50% sequence identity of the hyaluronidases in bee and vespid venom. However, a recent study revealed that the wasp hyaluronidase is only a minor allergen, and cross-reactivity between vespid and honeybee venom is not due to protein cross-reactivity, but is mainly caused by cross-reactive carbohydrate determinants (CCDs) [2]. Generally, CCDs are a frequent cause for double positivity as CCD-specific IgE (sIgE) mimics DS in vitro. Asparagine linked carbohydrate moieties of plant and insect glycoproteins are the structural basis of CCDs. In hymenoptera venom, these moieties are found in honeybee venom phospholipase A2 (Api m 1) and hyaluronidase (Api m 2), in vespid venom only in hyaluronidase (e.g. Ves v 2). CCD-sIgE is believed to be clinically irrelevant, although the underlying mechanisms are not completely understood [3], [4].

In cases of double positivity, also characteristics of different methods of serum IgE determination should be regarded: Depending on the method, frequencies of double-positive test results vary and range from 10 to 59% [1], [5]. In this context, affinity may play an important role. Affinity is largely determined by the stability of the allergen/IgE complex; therefore low affinity is usually correlated with a rapid dissociation of the complex. To efficiently activate mast cells or basophils, high affinity antibodies are required. Most of the current systems of IgE determination use high doses of allergen for IgE detection due to the binding competition with specific IgG. As a consequence low affinity IgE antibodies [6], which are thought to be less relevant for eliciting an allergic reaction [7], are bound as well. Nevertheless, low affinity IgE is not completely irrelevant: in the presence of high affinity IgE it may also activate basophils [8].

The intradermal test is considered not to be influenced by CCDs, as low affinity antibodies itself are not able to cause positive reactions. However, clinically irrelevant positive test results at 1,0 µg/ml are frequently observed [9] and side effects cannot be ruled out [10].

Several studies confirmed the usefulness of the CD63 based basophil activation test (BAT) as a routine diagnostic tool [11], [12], [13] and as a valuable test in patients with inconclusive tests and history (negative skin tests, undetectable sIgE or unknown stinging insect) [14], [15]. Compared with the IgE determination in the serum, BAT has the advantage of demonstrating functional responses: Positive test results will only occur after successful cross-linking of two identical FcεRI-bound IgE antibodies and not by monovalent binding like in IgE assays.

Recently, the component resolved diagnosis (CRD) has been described as useful tool to facilitate the diagnosis of bee and vespid venom allergy [1], [16]. Nevertheless, in these studies only rApi m 1 and rVes v 5 were employed to discriminate between true and CCD-based DS. But it is crucial to additionally determine Ves v 1, otherwise 10–13% of vespid venom allergic patients will not be diagnosed due to a mono-sensitization to Ves v 1 [1], [2].

Treatment of double positive patients with both venoms is a pragmatic way, but frequently not justified because of asymptomatic sensitization or cross-reactions caused by CCDs. Therefore there is still need for a test which is able to discriminate between clinically relevant or irrelevant sensitization in order to reduce the burden of treatment and to keep therapy as cost-efficient as possible.

In clinical routine, we observed a high frequency of double positivity in the IgE determination by the CAP system (ImmunoCAP®, Phadia, Uppsala, Sweden) and a markedly lower frequency of double positive results obtained by the BAT. Giving this background, we initiated a prospective study to evaluate the usefulness of new diagnostic approaches for the routine diagnosis of hymenoptera venom allergy. For this purpose, we aimed to compare the outcomes of the BAT with the IDT (intradermal test) as well as with three different methods of IgE determination (CAP, ADVIA (ADVIA Centaur®, Siemens, Tarrytown, NY, USA), Immulite (Immulite 2000®, Siemens, Tarrytown, NY, USA)) regarding the frequency of double positive results. To study IgE binding patterns, western blot (WB) inhibition as well as a CRD with native and recombinant Api m1, Ves v 1 and Ves v 5 were performed in all patients with DS.

## Methods

### Patients

One hundred and seventeen consecutive patients, who had been admitted to our outpatient clinic because of systemic allergic reactions with at least generalized skin symptoms after a hymenoptera sting, were screened. Their personal history was taken and the current standard diagnostic procedures (intradermal tests, IgE determination by CAP) were performed. As wasp and European hornet belong to the family of Vespidae and their venoms contain the same major antigens, we did not differentiate between these genera. Additionally, sIgE was determined by ADVIA, and the Immulite; basophil responsiveness was analyzed by a CD63 based BAT. In 72 patients showing specific IgE to honeybee and vespid venom in the CAP system, IgE patterns were determined by WB inhibition and CRD. This study was approved by the ethics committee of the Medical University of Graz.

### Personal history

According to the modified classification of Ring and Messmer, generalized skin symptoms such as flush, urticaria and angioedema were classified as grade I reaction. Mild to moderate pulmonary, cardiovascular or gastrointestinal symptoms were rated as grade II reaction. Bronchoconstriction, emesis, anaphylactic shock, and loss of consciousness were classified as grade III reaction.

### Reagents

All laboratory reagents were obtained from Merck (Whitehouse Station, NJ, USA) or Sigma-Aldrich (St Louis, CA, USA) unless otherwise specified. Dulbecco's modified phosphate-buffered saline (PBS; with or without Ca^2+^ and Mg^2+^) was purchased from Gibco-Invitrogen (Carlsbad, CA, USA). CellFix and anti-CD123 (PE-conjugated) were supplied by Becton Dickinson (Franklin Lakes, NJ, USA). Antibodies to HLA-DR (PC5-conjugated), CD63 (FITC-conjugated), and monoclonal antibodies to IgE were purchased from Beckman Coulter (Fullerton, CA, USA). Honeybee and vespid venom for the skin tests and BAT were purchased from ALK-Abelló (Hørsholm, Denmark). Honeybee venom and vespid venom sac extracts (mixture of *Vespula vulgaris* and *germanica*) were kindly provided by Vespa Laboratories, PA, USA.

### Skin tests

The nature of sensitization was confirmed by standardized end-point titration IDTs (0.02 mL of 0.001, 0.01, 0.1 and 1 µg/mL solution) using purified honeybee and vespid venom extracts. IDTs were considered to be positive in the presence of a wheal ≥5 mm in diameter and erythema.

### Determination of sIgE and tIgE

Specific and total IgE antibody levels in the patients' serum were measured using ImmunoCAP 1000 (Phadia, Uppsala, Sweden), ADVIA Centaur, and Immulite 2000 (both: Siemens, Tarrytown, NY, USA) according to the manufacturer's instructions. The CRD with native and recombinant nApi m 1 and rApi m 1 was done on the ImmunoCAP 1000. Diagnosis with the major wasp allergens nVes v 1 and nVes v 5 as well as with nApi m 1 was done on the ADVIA Centaur platform by the Department of I+D, ALK-Abelló, Madrid, Spain.

### Basophil activation test (BAT)

BAT was performed as previously described [17]. In brief, EDTA whole blood was stained with anti-CD123 PE-conjugated antibody (1∶50), anti-HLA-DR PC5-conjugated antibody (1∶50) and anti-CD63 FITC-conjugated antibody (1∶50). Basophil reactivity was measured using serial dilutions of honeybee or vespid venom (1000, 100, 10, 1 ng/mL) or serial dilutions of anti-IgE antibody (1∶10–1∶1000 dilution).

Finally, cell samples were analyzed by three-color flow cytometry (FC 500, Beckman Coulter). Basophils were identified as a single population of cells that stained positive for CD123 (FL-2) and negative for HLA-DR (FL-4). Up-regulation of CD63 expression was indicated by an increase in fluorescence in the FL-1 channel. Acquisition was terminated after 500 basophil target events. An approximately 2.5-fold increase in the number of activated basophils (>25%) as compared with the negative control (10%) at any of the test concentrations of the allergen was considered to be a positive response. This threshold was determined by ROC analysis as described earlier [12].

### Western blots and western blot inhibition

Honeybee venom and vespid venom were separated by SDS-PAGE using 13.5% resolving and 5.7% stacking gels under reducing conditions using dithiothreitol and heat. Electrophoretically separated proteins were blotted onto nitrocellulose membranes and single strips (6 µg venom/strip) blocked with PBS buffer (50 mM sodium phosphate, pH 7.5, 0.5% Tween 20, and 0.05% NaN_3_) containing 0.5% BSA at room temperature for 1 h. Subsequently, strips were incubated overnight with 1 mL of serum (diluted 1∶5–1∶10) at 4°C under continuous shaking. After washing twice with PBS buffer for 30 min, bound IgE was detected by ^125^I-labelled rabbit anti-human IgE (Phadia, Uppsala, Sweden). After overnight incubation at room temperature, washed and dried strips were exposed to a high-performance autoradiography film (Hyperfilm MP, Amersham, England) at −70°C for 5–10 days.

To discriminate between IgE specific for peptide or carbohydrate epitopes, antibody binding to CCDs was inhibited by preincubating sera with 5 µg/mL of MUXF-BSA as done in previous studies [18]. MUXF-BSA is a synthetic glycoprotein obtained by coupling purified N-glycans from pineapple stem bromelain to BSA [19], whereby MUXF (or more exactly MUXF^3^) stands for the glycan structure Manα1-3(Xylβ1-2)Manβ1-4GlcNAcβ1-4(Fucα1-3)GlcNAcβ1.

### Data analysis

All data are expressed as medians (25%; 75% percentiles) on the raw scale, unless otherwise indicated. Data were tested for normality using the Kolmogorov-Smirnov test. Continuous variables were analysed by the Kruskal Wallis test; categorical variables were compared by the Chi-square test or Fisher's exact test. To check agreement between the tests, Cohen's kappa coefficient was calculated. The level of significance was set at p<0.05. The SPSS 17.0 software (SPSS Inc, USA) was used for statistical analysis.

## Results

### History and demographic data

One hundred and seventeen patients with a unequivocal history of a systemic sting reaction were included in the study. Fifty-eight (49.6%) were female, and 59 (50.4%) male. Median age was 42.0 (30.5; 53.0) years; the majority of patients (45.3%) were in the age group between 30 and 50 years.

Four patients (3.4%) had a history of grade I reactions, 80 patients (68.4%) had experienced grade II reactions and 33 patients (28.2%) grade III reactions. Thirty-eight (32.5%) identified a honey bee as culprit insect, 55 (47.0%) a wasp, and 24 (20.5%) could not identify the insect. None of the patients reported systemic sting reaction after both, honeybee and wasp stings.

### Double sensitization

**Figure 1:**
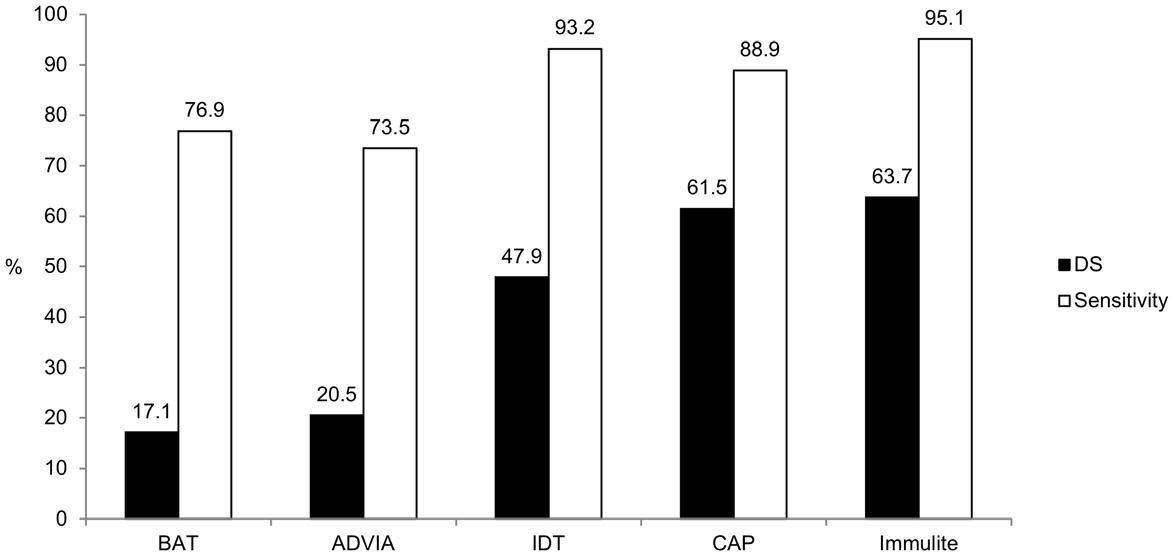
Frequency of double sensitization. The rate of DS in 117 consecutive patients differed significantly (p<0.0001) and ranged from 17.1% with the BAT to 63.7% with the Immulite.

Frequency of DS differed considerably among performed diagnostic tests and ranged from 63.7% with the Immulite to 17.1% with the BAT (Figure 1). Generally, agreement of tests was fair with 53.1% (kappa 0.318; p<0.0001)

### Differences between mono and double sensitized as well as double negative patients

In all tests except in the BAT, tIgE levels were up to 2.3-fold higher in double sensitized patients compared to mono sensitized patients. Conversely, patients with double negative results had lower tIgE levels compared to mono or double sensitized patients (Table 1). The comparison of mean age between the three categories revealed no significant difference.

**Table 1:** Correlation between total IgE (kU/L) and test results.

	Double sensitization	Mono sensitization	Double negative	p
BAT	54.3 (24.5; 217.3)	64.7 (36.9; 151.0)	58.9 (22.7; 112.0)	0.463
ADVIA	117.0 (50.9; 397.6)	51.7 (30.3; 123.4)	35.9 (10.4; 142.4)	0.008
IDT	88.7 (45.2; 252.0)	53.9 (29.4; 103.5)	35.3 (8.7; 69.0)	0.014
CAP	90.8 (48.9; 230.0)	43.3 (29.1; 64.2)	28.0 (10.6; 66.0)	0.000
Immulite	87.2 (42.6; 246.0)	51.5 (19.6; 87.5)	8.0 (2.7; 95.6)	0.006

In all tests except in the BAT, double sensitized patients showed higher levels of total IgE (tIgE) compared to mono sensitized patients. Conversely, double negative patients had lower tIgE levels compared to mono- and double sensitized patients.

Additionally, regression analysis to check the influence of the severity of sting reaction, sex, age and tIgE on DS was performed: The frequency of DS was influenced by tIgE levels in the CAP (e^b^ 1.005, p = 0.035) and ADVIA (e^b^1.003, p = 0.048). Additionally, higher age of the patients was associated with a lower frequency of DS in the CAP (e^b^ 0.966, p = 0.038). The rate of DS in the BAT, IDT, and Immulite was not influenced by the tested variables.

### Subgroup analysis of double sensitized patients in the CAP

IgE determination by CAP yielded together with the Immulite the highest frequency of double positive results. As the CAP system is widely used, and this group of 72 patients comprised virtually all patients with double positive results in supplemental tests, further analysis regarding the individual IgE pattern was done in this subgroup.

**Figure 2:**
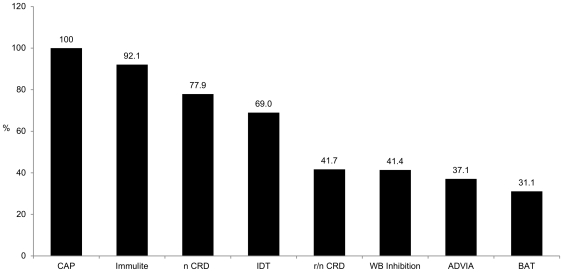
Frequency of double sensitization in supplemental tests in 72 CAP double positive patients. n CRD: native component resolved diagnosis with nApi m 1, nVes v 1, nVes v 5. r/n CRD: combined component resolved diagnosis with recombinant rApi m 1, and native nVes v 1, nVes v 5. BAT (p = 0.324) and ADVIA (p = 0.874) showed a similar frequency of DS compared to WB inhibition and r/n CRD, although they were performed with native venom extracts.

First, the rate of DS of each commercially available and experimental test was determined to identify the most specific test to reduce the high frequency of clinically not relevant DS. As expected, CRD analysis solely done with the native main allergen components nApi m 1, nVes v 1, and nVes v 5 led to a slightly reduced, but still high frequency of DS. The use of non-glycosylated rApi m 1, nVes v 1 and nVes v 5 reduced the frequency considerably by 49.0%. Similar lower rates of DS were observed with the WB inhibition, ADVIA and BAT, while the Immulite and the IDT revealed high frequencies of DS (Figure 2).

### IgE patterns of CAP double sensitized patients with WB inhibition

**Figure 3:**
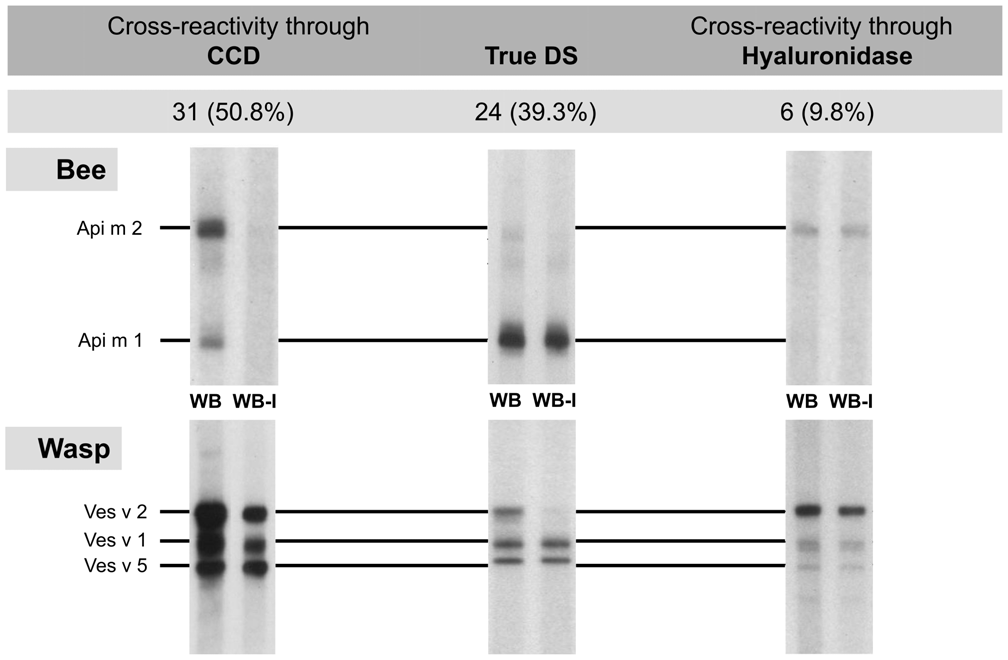
Frequency of typical IgE patterns obtained by western blot inhibition in CAP double sensitized patients. CCD: cross-reactive carbohydrate determinants, True DS: true double sensitization, WB: western blot, WB-I (western blot inhibition): To discriminate between IgE specific for peptide or carbohydrate epitopes, antibody binding to CCDs was inhibited by preincubating sera with MUXF-BSA. Among these patients the majority of DS was CCD-dependent. DS due to protein components of hyaluronidases played a minor role. n = 61.

The WB was not interpretable in 11 of 72 patients. Among the remaining patients, true DS was diagnosed in 24 of 61 patients, putative cross-reactivity due to hyaluronidase in 6 patients, and double positive results caused by CCD alone in 31 patients (typical IgE patterns see Figure 3).

### CRD in CAP double sensitized patients

>As at the time when the study was performed rApi m 1 was not available for the ADVIA, and vice versa nVes v 1 and nVes v 5 not for the CAP, rApi m1 was determined with the CAP and nVes v 1 and nVes v 5 with the ADVIA. To check compatibility, nApi m 1 was determined on the CAP as well as ADVIA. In contrast to the IgE determination with bee and vespid extracts, the test results with native components were coinciding with 92.3%, assuming an almost perfect agreement.

**Figure 4:**
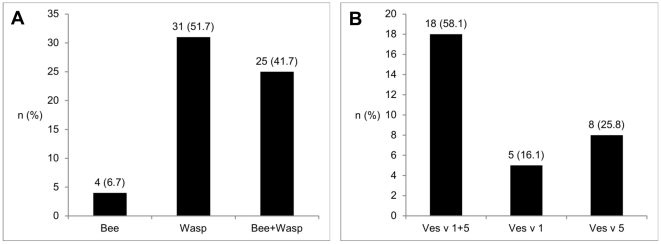
Component resolved diagnosis in CAP double sensitized patients. **A Sensitization to bee and/or vespid venom in the component resolved diagnosis**. Positive for bee venom: rApi m 1^pos^ / nVes v 1^neg^ and nVes v 5^neg^; Positive for wasp venom: rApi m 1^neg^ / nVes v 1 and/or nVes v 5^pos^; DS: rApi m 1^pos^ / nVes v 1 and/or nVes v 5^pos^. n = 60. **B Sensitization pattern in vespid venom allergic patients**. The majority of patients were sensitized to both vespid major allergens (nVes v 1 and nVes v 5). Nevertheless, a considerable proportion had a mono sensitization to nVes v 1 or nVes v 5. n = 31.

Finally, CRD with recombinant and native allergens was performed in 64 of 72 CAP double positive sera; four patients were negative for the tested bee and vespid venom allergens (Figure 4 A+B). There was a substantial agreement between the WB and the CRD for Api m 1 with 88.5% (kappa 0.770, p<0.0001), and Ves v 5 with 87.7% (kappa 0.744, p<0.0001). The agreement for Ves v 1 was only fair with 71.9% (kappa 0.377, p = 0.005).

### BAT compared to CRD and WB inhibition in CAP double sensitized patients

**Figure 5:**
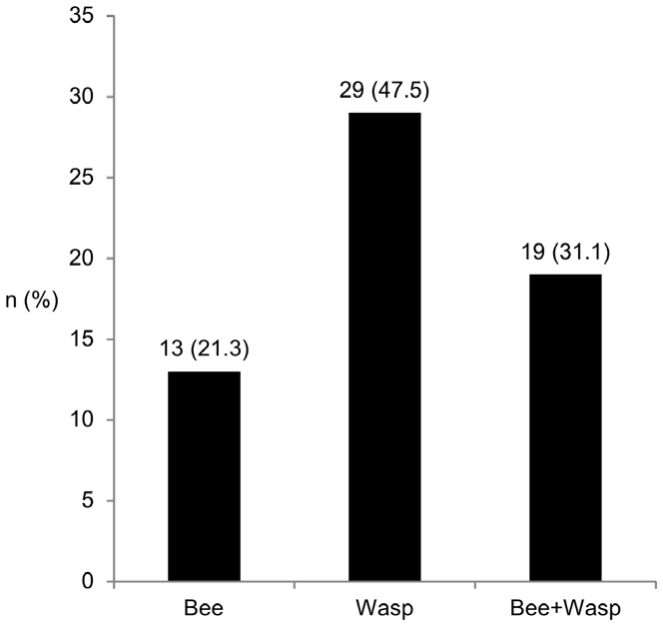
Frequency of sensitization to bee and/or vespid venom in the basophil activation test. n = 61.

**Figure 6:**
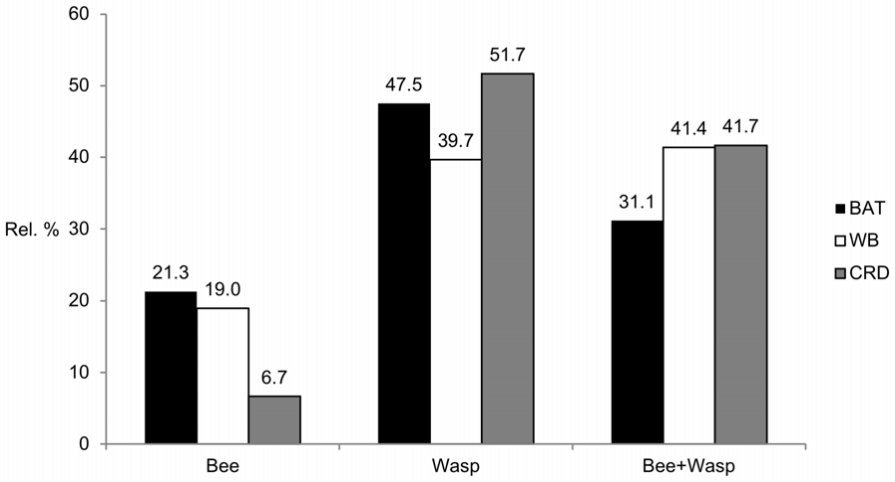
BAT results in relation to western blot inhibition and component resolved diagnosis. Although BAT was performed with native venom extracts, frequency of mono- and double sensitization was comparable with component based methods. Results of the BAT were in fair agreement with those of the CRD (60.0%, kappa 0.373, p<0001) and WB (59.6%, kappa 0.377, p<0001). Interestingly, the frequency of honey bee sensitization obtained with the CRD was markedly lower compared to BAT and WB, which could indicate a lower sensitivity of rApi m 1.

Beside the component-specific tests (CRD, WB) only the BAT and ADVIA showed a comparable low frequency of DS despite the use of conventional allergen extracts. As ADVIA is no longer available, further analysis was only done with the BAT in 72 patients; 11 were negative for both venoms (Figure 5). Noteworthy, in 11 patients with DS in the CRD, basophils were only activated by one venom in the BAT. Conversely, 7 BAT double positive patients showed only a mono sensitization in the CRD. There was a similar picture with the WB inhibition: 13 double positive patients in the WB inhibition were only positive to one venom in the BAT and 7 BAT double positive patients showed only a mono sensitization in the WB inhibition. Generally, results of the BAT were in fair agreement with those of the CRD (Figure 6).

### sIgE to MUXF (CCD)

**Figure 7:**
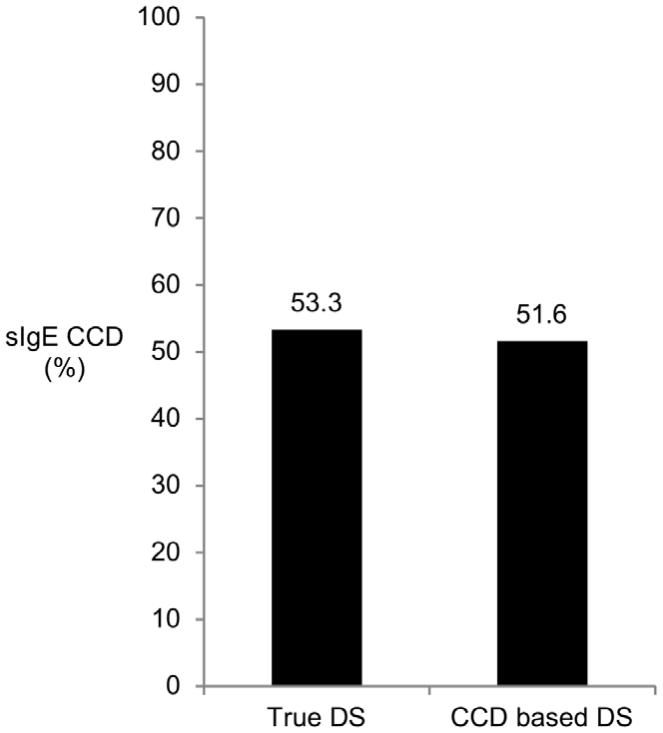
Determination of sIgE to MUXF (CCD) was not appropriate to distinguish between true and CCD-based double sensitization. Patients with true DS in the WB (sensitization to major allergens or hyaluronidase) had detectable sIgE to MUXF and conversely, patients with a verified CCD-based DS by the WB inhibition had no detectable sIgE to MUXF. As the coincidence of true DS and detectable sIgE to MUXF was high, results could be misinterpreted and true DS could be easily overlooked.

Determination of sIgE to MUXF in the CAP (CCD-IgE) was not appropriate to distinguish between true DS and CCD based DS. 16 of 30 patients with true DS in the WB (sensitization to major allergens or hyaluronidase) had detectable sIgE to MUXF and conversely, only 16 of 31 patients with a verified CCD-based DS by the WB inhibition had sIgE to MUXF (Figure 7).

Additionally, also 15 of 25 (60.0%) patients with true DS verified by CRD had sIgE to MUXF.

### Double positive results in CCD-dependent DS

**Figure 8:**
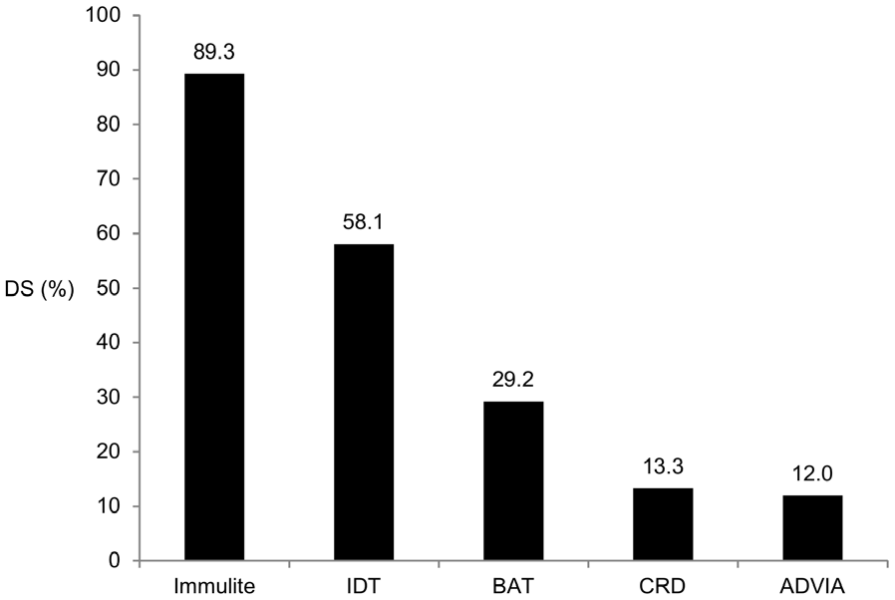
Double positive results in CCD-dependent double sensitization. CCD-dependent DS was verified with WB inhibition in 31 patients. The Immulite and IDT revealed the highest rates of DS in these patients (p<0.001; n = 24–31).

CCD dependent DS was verified by WB inhibition in 31 patients. Depending on the test, frequency of DS ranged from 12.0% to 89.3% in these patients (Figure 8).

## Discussion

Positive test results to bee and vespid venom are frequently observed in the routine diagnosis of hymenoptera venom allergy and raise problems to determine the causative insect for a correct treatment. Treatment with two venoms is generally accepted in patients with severe sting reactions and inconclusive test results. Nevertheless, there is a high risk of overtreatment, and even for a novel sensitization, if positive results are unspecific and caused by weakness of diagnostic methods or by CCDs.

In the current study, we performed an extensive evaluation of various conventional, recently established, and experimental test methods. We could demonstrate that the BAT had the lowest frequency of DS and thus correlated best with the patients' history. Nevertheless, the BAT showed double positive results in nearly one third of patients with CCD-based DS, and vice versa was sometimes only positive for one venom in patients with DS in the WB inhibition and CRD. CCDs can lead *in vitro* to a stimulation of basophils [20], [21] and the question of clinical relevance of these positive results remains still unanswered. Conversely, even a true (double-) sensitization must not be clinically relevant [22], [23]. In this case, the BAT as functional test may be helpful to find the culprit venom. IgE determination by the ADVIA also resulted in a low frequency of DS, even though it was slightly higher compared to the BAT. However, the ADVIA platform is no longer available for routine diagnosis as it has been taken off the market despite of its revolutionary concept of IgE determination and its excellent performance. Additionally, we could show that the intradermal test was not beneficial in the discrimination between mono- and double sensitization because it revealed DS in as much as 69% of patients. This may either reflect false-positive reactions due to histamine liberating substances or toxic effects of the venom, as well as some mast cell activation by CCDs at very high venom concentrations (1 µg/ml). As expected, the CRD with recombinant and native CCD-free allergens discriminated well between CCD based and true DS, and hence represents a clear step forward in the diagnosis of hymenoptera venom allergy. Importantly, the sensitization patterns of the CRD correlated well with those of the western blot. Nevertheless, the CRD revealed a markedly lower frequency of honey bee sensitization compared to BAT and WB which could indicate an insufficient sensitivity of rApi m 1 and the need for additional honeybee venom allergens.

Clinically relevant DS is rarely observed: in a large European (EAACI) multicenter study regarding side-effects during immunotherapy only 58 of 840 (6.9%) were treated with two venoms [24]. At the same time, asymptomatic sensitization is observed in 27.1 to 66.7% of the general population depending on the test method of IgE determination and tIgE levels [5], [23].

Depending on the methods and venoms used, the specificity of serum IgE determination ranges between 60% and 94% [12]. Leading manufacturers of automated lab systems generally postulate high sensitivities and specificities for their IgE determination. However, the studies leading to these results must be viewed critically: control subjects with high tIgE levels, positive skin tests and an atopic disposition are generally ruled out in order to obtain optimum specificities [25], [26].

Generally, methods of serum IgE determination differ considerably and therefore results are difficult to compare. In CAP, the allergen is bound to a solid cellulose sponge matrix. After incubation with the patient's serum sIgE and also specific IgG is bound to the covalently coupled allergen. To quantify sIgE levels, sIgE is detected by enzyme-labeled anti-IgE. To minimize competition between the low quantity of IgE and the substantial quantity of IgG a very high amount of allergen is bound to the immunosorbent. Therefore also low-affinity cross-reacting sIgE like those to CCDs with questionable clinical relevance are detected. The same might be valid for the Immulite, although it depends on another principle: In brief, ligand-labeled liquid allergens first bind to anti-ligand-coated polystyrene beads; after adding the patient's serum, sIgE is bound to the allergen. Again, sIgE is detected by anti-IgE. High doses of allergen to avoid displacement of sIgE antibodies in both tests would explain the similar frequency of DS with 61.5% and 63.7%, respectively.

The concept of the ADVIA is completely different to exclude interference with non-IgE antibodies like IgG. Anti-IgE is coupled to paramagnetic particles that catch all IgE in the serum. Then biotin-labeled allergen is added and bound sIgE reacts with the allergen in suspension. Finally sIgE is detected indirectly with acridinium ester labeled streptavidin [27]. The main advantage of this approach is that much less allergen is needed and therefore the affinity of sIgE is better considered. This explains the good performance of the ADVIA despite of the native venom extracts used.

The IDT and the BAT have the advantage of demonstrating functional responses as positive results usually only occur after cross-linking of two identical cell-bound IgE antibodies. Nevertheless, we observed a considerable difference in the occurrence of DS: The IDT was positive for bee and vespid venom in 47.9% of patients compared to 17.1% double positive results obtained by the BAT. The high frequency in the IDT might be explained by the irritant effect of the venom at higher doses and, as mentioned earlier, by the activation of some mast cells by CCDs at very high venom concentrations. On the other hand, the low rate of DS in the BAT with native venom extracts supports the hypothesis, that the BAT is able to demonstrate a functional response without possible irritant reactions as seen in the IDT and without considerable influence of CCDs on test results as obtained with the CAP or Immulite. Recently, up to 67% double positive results were reported with the CD203c based BAT [28], this is contrary to our findings. This extraordinary high rate of DS might not depend on the different activation marker CD203c, but on an internationally uncommon protocol and unusual interpretation of results. Nevertheless, there still remain a few open questions: In our study, the BAT showed in 29% of patients with a verified CCD-based DS double positive results and vice versa the BAT was sometimes only positive for one venom despite that the CRD and WB inhibition revealed double positive results, respectively.

The role of CCDs for eliciting clinical symptoms is still unclear. There are several hypotheses why sIgE to CCDs are not relevant, one of them is that patients are constantly exposed to these carbohydrate structures and therefore produce blocking IgG_4_ antibodies, comparable with the effect of immunotherapy [4]. This might explain that basophils can be activated in the BAT, but not in vivo.

The application of recombinant or native CCD-free allergens will be a considerable progress in the diagnosis of hymenoptera venom allergy. nApi m 1 showed clearly more positive results compared to rApi m 1, again indicating the crucial role of CCDs in DS. Thus makes it inevitable to use components which are CCD-free by nature or to produce recombinant allergens without CCDs. Importantly, the generally accepted use of sIgE to CCD as marker for CCD-based cross-reactivity has to be viewed critically and must be considered obsolete. As shown in the WB, the presence of IgE to CCDs does not exclude true DS, therefore true DS can be easily overlooked, which may result in fatal reactions.

To summarize, BAT and CRD showed the lowest rates of DS, but inconsistent results were common. Although each test alone seems to help finding the clinically relevant venom, it is still unclear which test represents the most accurate. Therefore, studies with sting challenges to check the accurate negative predictive value of the BAT and CRD in otherwise double sensitized patients would be preferable. At present, no routinely employed test can be regarded as gold standard to distinguish between clinically relevant bee and wasp venom sensitization.
